# Digitalized Human Organoid for Wireless Phenotyping

**DOI:** 10.1016/j.isci.2018.05.007

**Published:** 2018-05-31

**Authors:** Masaki Kimura, Momoko Azuma, Ran-Ran Zhang, Wendy Thompson, Christopher N. Mayhew, Takanori Takebe

**Affiliations:** 1 Division of Gastroenterology, Hepatology & Nutrition, Developmental Biology, Center for Stem Cell and Organoid Medicine (CuSTOM), Cincinnati Children's Hospital Medical Center, 3333 Burnet Avenue, Cincinnati, OH 45229-3039, USA; 2 Department of Pediatrics, University of Cincinnati College of Medicine, 3333 Burnet Avenue, Cincinnati, OH 45229-3039, USA; 3 Institute of Research, Tokyo Medical and Dental University, 1-5-45 Yushima, Bunkyo-ku, Tokyo 113-8510, Japan; 4 Advanced Medical Research Center, Yokohama City University Graduate School of Medicine, Kanazawa-ku 3-9, Yokohama, Kanagawa 236-0004, Japan

**Keywords:** Cell Biology, Stem Cells Research, Bioengineering

## Abstract

Radio frequency identification (RFID) is a cost-effective and durable method to trace and track individual objects in multiple contexts by wirelessly providing digital signals; RFID is thus widely used in many fields. Here, we implement this concept to biological tissues by producing a compact RFID chip-incorporated organoid (RiO). The 0.4 mm RFID chips are reproducibly integrated inside the self-assembling organoids from 10 different induced pluripotent stem cell (iPSC) lines from healthy and diseased donors. We use the digitalized RiO to conduct a phenotypic screen on a pool of RiO, followed by detection of each specific donor *in situ*. Our proof-of-principle experiments demonstrated that a severely steatotic phenotype could be identified by RFID chip reading and was specific to a genetic disorder of steatohepatitis. Given evolving advancements surrounding RFID technology, the digitalization principle outlined here will expand organoid medicine potential toward drug development, precision medicine, and transplant applications.

## Highlights

• Generation of radio frequency identification (RFID) tag integrated human organoids
• Self-assembly culture enables RFID-integrated organoids with intact functions
• Microscopic phenotypic assay coupled with donor identification by RFID
• Proof-of-principle of organoid digitalization with RFID for steatosis screening

## Graphical Abstract

**Figure:**
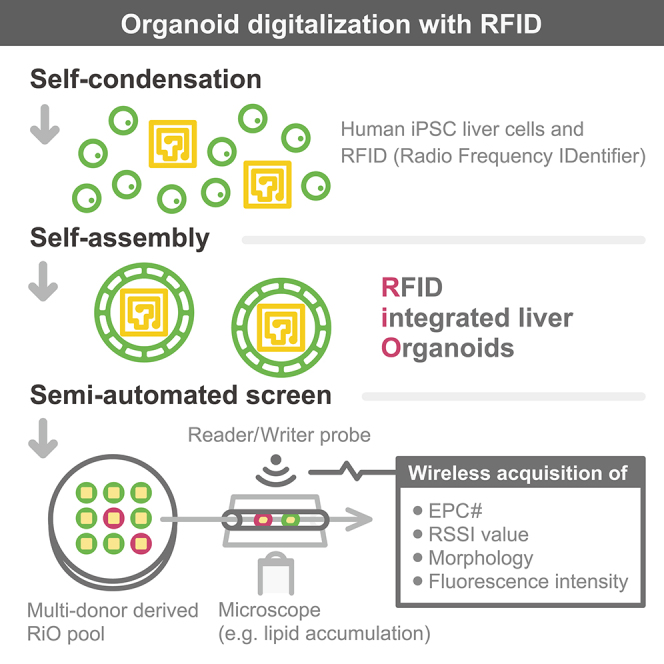

## Introduction

Radio frequency identification (RFID) is a cost-effective technology for addressing personal identification, traceability, and environmental considerations, especially in transportation industries, which has permeated all facets of modern life (Finkenzeller, 2010, Pardal and Marques, 2010, Want, 2006). RFID tags, operating wirelessly, collect energy from a nearby reader's interrogating radio waves. The high degree of tolerance to tested solutions, solvents, extreme temperatures, and high- or low-pressure conditions (Leung et al., 2010) provides the RFID tags with significant advantages over barcodes. The tags are now integrated into cards, clothing, and possessions, as well as implanted into animals and humans. For example, RFID tags are incorporated into cards and are used to pay for mass transit fares on buses, trains, and subways and to collect tolls on highways in many countries. In 2017, the world RFID market, which includes tags, readers, software/services for RFID cards, labels, fobs, and all other form factors, was worth US$11.2 billion and has an estimated 10% annual growth, resulting in an anticipated value of US$18.68 billion by 2026 (Das, 2017).

In recent years, there has been considerable interest in extending the usage of RFID to the healthcare arena. For example, implanting RFID microchips in animals and humans allows for positive identification of specific individuals. Medical application of RFID now includes an oral “digital pill” for chronic conditions, in efforts to improve patients' adherence (Chai et al., 2015, Chai et al., 2016). The ingested radiofrequency emitter, once activated by gastric pH, emits a radiofrequency signal, which is captured by a relay hub and transmitted to a smartphone, where it provides ingestion data and deliver interventions in real time. Thus, the diverse applications of RFID provide innovative solutions to various biomedical challenges. Similarly, RFID incorporation into cells or tissues can provide advancements in tracking *in vitro* and *in vivo* processes, in drug discovery, and in understanding disease mechanisms. Recent applications of micro RFID showed the passive intra-cellular delivery and short-term persistence; however, the use of mouse phagocytic cell line and melanoma cell line limit its broader application (Hu et al., 2017). Thus, realistic RFID applications in the tissue culture context necessitates viable methods to incorporate the microchip into biological tissue without impairing the tissue's native structure and functions.

Recently, human organoids have received international attention as an *in vitro* culture system where human stem cells self-organize into three-dimensional (3D) structures reminiscent of human organs (Lancaster and Knoblich, 2014, Takebe et al., 2013, Takebe et al., 2015). Organoids, owing to their higher phenotypic fidelity to human disease (Workman et al., 2017), are expected to provide a mechanistic assay platform with future potential for drug screening and personalized medicine (Clevers, 2016, Dutta et al., 2017, Sachs et al., 2018). It becomes feasible to study human pathological variations by comparing genotypes with phenotype spectrum diseases, including cystic fibrosis (Saini, 2016), steatohepatitis (Takanori Takebe et al., Unpublished), and cholestatic disease (Takanori Takebe et al., Unpublished), using a human induced pluripotent stem cell (iPSC) or adult stem cell library. One key feature of organoids is the development of a polarized structure surrounded by basement membrane through a self-assembly process, resulting in a cavitated structure from an aggregated tissue. Therefore, we hypothesized that aggregation-mediated self-assembling process will enable the successful internalization of miniature chips into biological tissues without compromising the native functions of the tissues.

**Figure 1:**
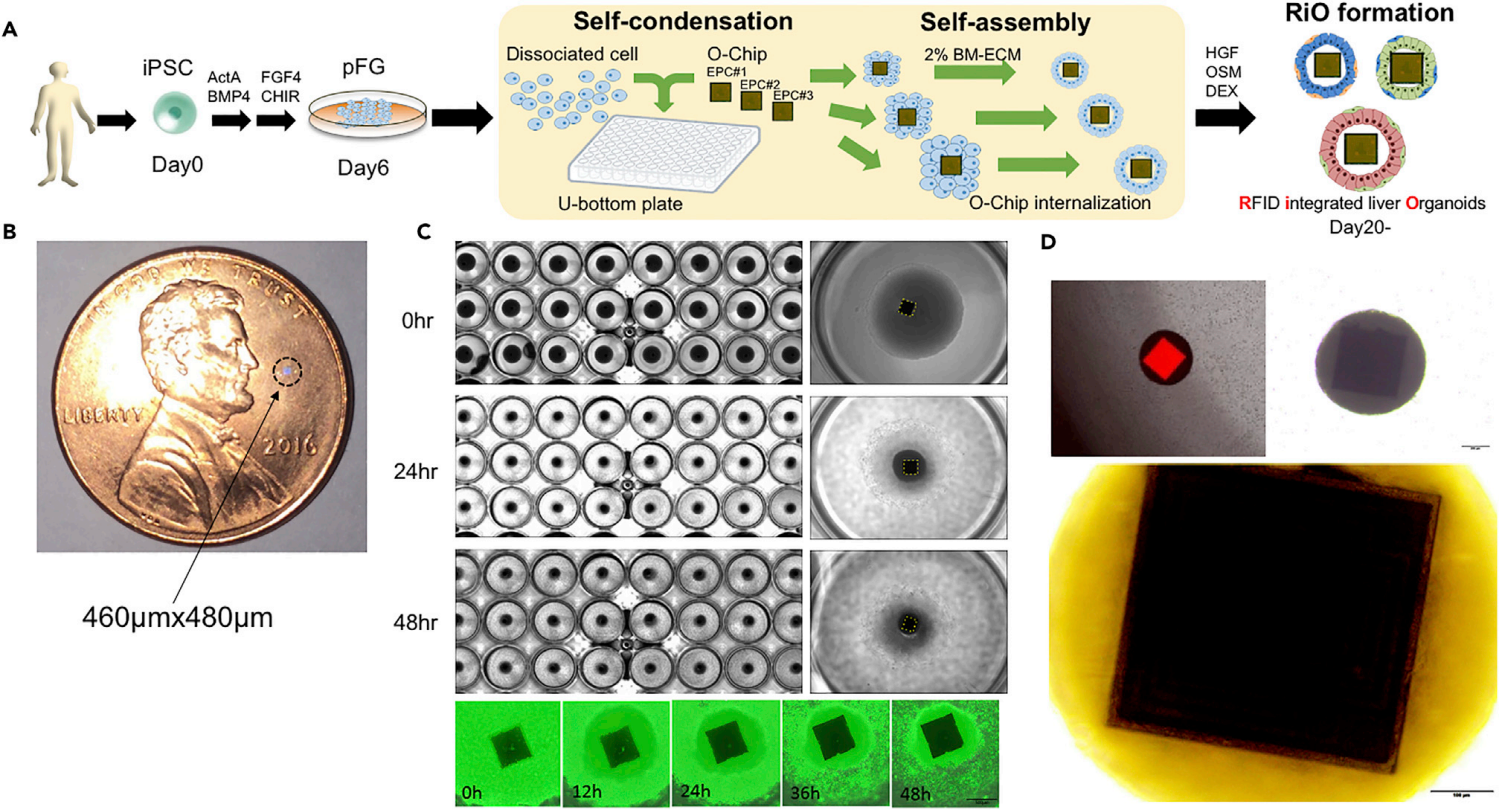
Concept of Organoid Digitalization with O-Chip (A) A schematic of organoid digitalization strategy. Integration of O-Chip into organoids makes it possible to digitalize organoids. (B) The size of the O-Chip: 0.46 ×0.48 μm^2^. (C) Self-condensation culture with O-Chip (Takebe et al., 2015). Serial pictures show that O-Chips are being integrated into organoids formed from iPSC derivatives. (D) RiO morphology. Each organoid completely encompasses one RFID microchip. Scale bars for RiO, 200 μm; for zoomed-in images, 100 μm.

Herein, we test this hypothesis by integrating ultracompact RFID chips into re-aggregated iPSC-derived endoderm spheroids before self-assembly. Recent advancements in miniaturization have generated ultra-compact RFID microchips ranging in size from 10 to 600 μm (Burke and Rutherglen, 2010, Chai et al., 2016, Chen et al., 2013). For simplicity and accessibility, we used commercially available organoid-scale RFID chips, herein defined as the O-Chip. The concept of organoid digitalization with O-Chip is shown in Figure 1A. Each O-Chip is 460 × 480 μm and has a 512 bit memory area (Figure 1B). By applying a specific wavelength into a coiled antenna, each O-Chip receives data sent from the reader/writer, and with energy driven by this electric current the O-Chip wirelessly sends information stored in its memory. The O-Chip operates wirelessly from readers across distances of up to about 1–2 mm.

## Results

**Figure 2:**
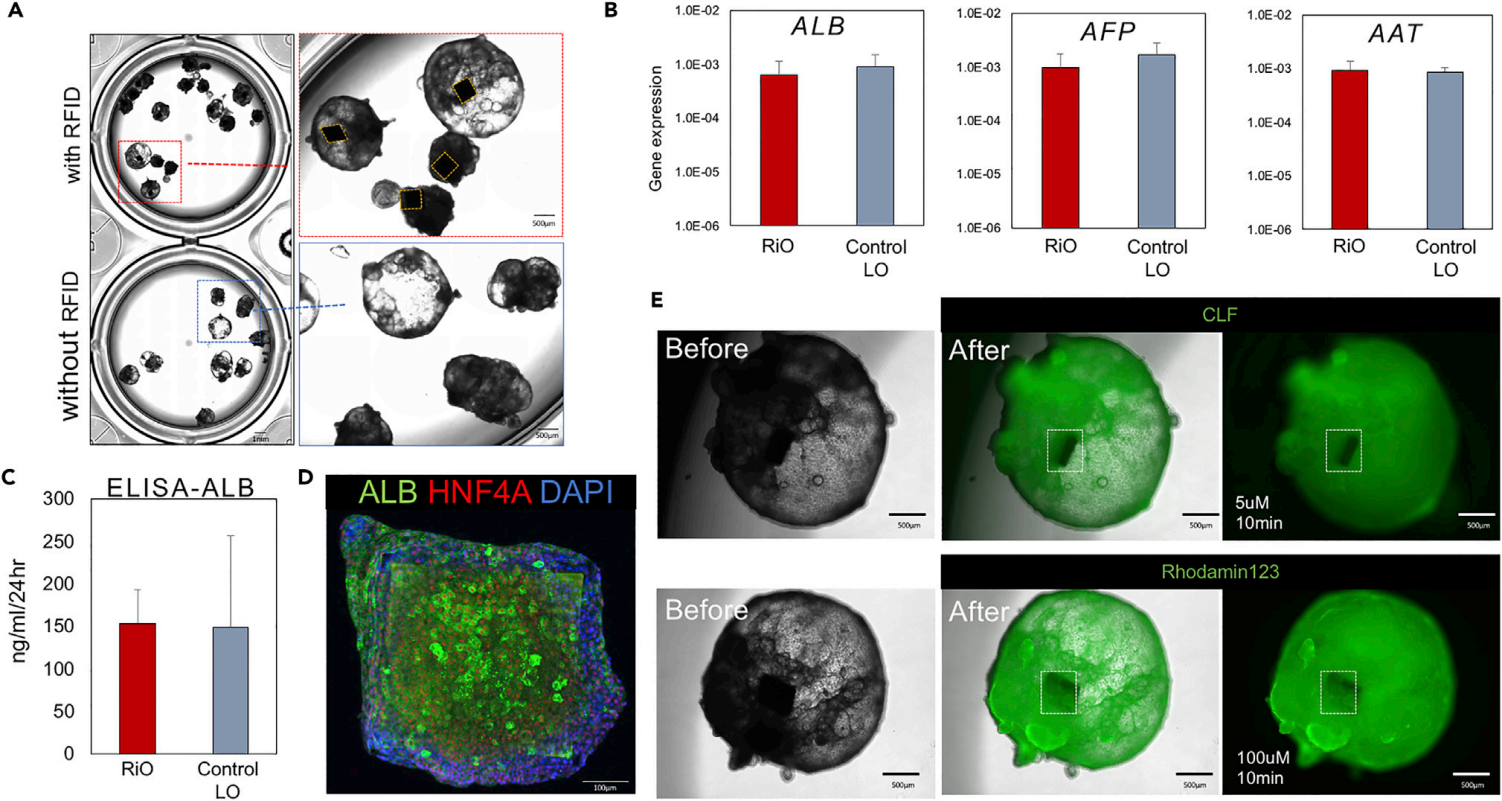
O-Chip Incorporation into Human iPSC Liver Organoids (A) A comparison between RiO and control LO demonstrating no differences in morphology. Scale bars for RiO, 1 mm; for zoomed-in images, 500 μm. (B and C) A comparison between RiO and control LO demonstrating no functional differences. (B) Gene expression analysis was performed for the liver-specific markers ALB, AFP, and AAT in RiO and control LO. (C) An ALB ELISA was performed on the supernatant collected from RiO and control LO. (D) A representative image of immunostaining for ALB, HNF4A, and DAPI on a RiO. Scale bars for RiO, 100 μm. (E) CLF and rhodamine123 uptake into RiO. Images were taken 10 min after each fluorescein exposure. Rectangle indicates O-Chip. Scale bars for RiO, 500 μm.

To test O-Chip integration into biological tissues, human iPSCs were initially differentiated into posterior foregut organoids by sequential Activin and FGF4/CHIR 99021 exposure. At day 6, foregut organoids were dissociated and seeded into 96 well plates, followed by O-Chip plating. Once re-aggregated tissues were formed, tissues were treated with a 2% laminin-rich basement membrane matrix to induce self-assembly or polarization. After 3 days in culture, RFID chip-incorporated organoids (RiOs) (Figures 1C and 1D) covered by hepatic epithelial cells were successfully generated (Figure 2A). We confirmed reproducible RiO generation by using eight different donor-derived iPSC lines (Figures S1A and S1B). The RiO formation efficiency, which means incorporation of RFID into the organoid, was very high and was 95% or more (92 of 96 organogens succeeded). Collectively, the O-Chip can be efficiently integrated inside iPSC-derived organoids.

To compare the RiO with standard liver organoids (control LO), a comparison of their morphology was first performed, and no morphological abnormalities in RiO were observed compared with control LO (Figure 2A). Next, we performed a qPCR analysis to detect liver-specific genes (Figure 2B). qPCR analysis revealed that RiO expressed equivalent levels of albumin (ALB), alpha-fetoprotein (AFP), and alpha-1-antitrypsin(AAT) compared with control LO (Figure 2B). To confirm these findings, we performed an ELISA of culture supernatant for the liver-specific protein ALB and found comparable protein levels between the RiO and control LO (Figure 2C). We found that the RiO secreted ALB at levels of 152.9 ng/mL after 24 hr, which is very similar to that of control LO (1.02-fold) (Figure 2C). Immunofluorescent whole-mount staining confirmed the presence of ALB+ cells inside the RiO (Figures 2D and S1C). Another liver marker, HNF4a, was found in the nucleus of ALB+ cells (Figures 2D and S1C).

To further investigate the functional capacity of RiO, we analyzed bile transport and fat accumulation capacity of RiO. The bile transport capacity of RiO was studied using the fluorescent bile acids cholyl-lysyl-fluorescein (CLF) and rhodamine 123, which are the substrates for the bile salt efflux pump (BSEP) and the cholangiocyte surface glycoprotein multidrug resistance protein-1 (MDR1), respectively. We have previously shown that control LO has the capacity to uptake CLF and rhodamine123 inside the organoids through BSEP and MDR1. Consistent with this, CLF and rhodamine123 were absorbed into the cells of RiO within minutes of the addition of the fluorescent bile acids. The fluorescent acids were then excreted and accumulated in the lumen of RiO (Figure 2E). The fat accumulation capacity of RiO was studied using the fatty acid treatment and lipid dye BODIPY 493/503 for lipids. We treated the RiO with fatty acids and then visualized the amount of lipid accumulation in the RiO using BODIPY (Figure S2). For quantification, we used ImageJ LUT to convert the BODIPY intensity in each image from brightness and darkness levels to a numerical value. In addition, we also analyzed iron accumulation in RiO (Figure S3). It was studied using the ammonium iron sulfate (FAS) treatment and Fe dye FeRhoNox 540/575. Thus, RiO possesses multiple human hepatic functions similar to control LO, including ALB secretion and bile transport function, suggesting that the presence of the RFID chip in the liver organoids does not seem to affect native structure and function.

RFID chips are generally highly durable, but their durability in multiple tissue culture contexts has not been examined. To determine their cryopreservation potential under ultralow temperatures, we examined the tolerance of O-Chip to freezing or to submersion in liquid nitrogen. For slow freezing and vitrification, O-Chip may be subjected to low temperatures during storage. We, therefore, tested a range of temperatures, from 4°C to −196°C, to determine tolerance and whether efficacy was affected (Table S1). In addition, during culture conditions the incorporated O-Chip must also endure dynamic changes in pH. Throughout exposures to these conditions, the RFID tags remained intact and durable, suggesting a high degree of tolerance to low temperatures and pH (Table S1). Moreover, the O-Chip remained functional after routine laboratory sterilization processes, such as autoclaving (dry and heat methods). The O-Chip incorporated in paraffin-embedded RiO could still be detected wirelessly. Since the paraffin embedding process required immersion of the O-Chip in water-based solutions and ethanol, these results supported tolerance of the O-Chip to solutions and solvents (Figure S4). The wireless detection of RiO-derived signals can be possible both in *in vitro* and *in vivo* settings (Figure S5). Collectively, O-Chips and RiO function robustly under a variety of environmental exposures routine to tissue culture protocols.

The remarkable durability of the O-Chip prompted us to test the cryopreservation potential of RiO. To develop an efficient method for optimal cryopreservation of RiO, we carried out comparative experiments using several different reagents and freezing methods (Figures S6A and S6B). The morphology of RiO was fully preserved after thawing after the gradual freezing method (Figure S6A), and the O-Chip could be read without being affected. To evaluate the potential for a phenotypic screening assay after freezing and thawing, we examined the previously frozen organoids by inducing steatosis according to unpublished protocols. The thawed RiO was exposed to free fatty acid, and BODIPY live-cell staining was performed to detect lipid accumulation. Fluorescence microscopy imaging showed that the cryopreserved RiO had retained the capability for accumulating lipid droplets (Figure S6D). Taken together, cryopreserved RiO not only retained its morphology but also preserved lipid storage functions.

**Figure 3:**
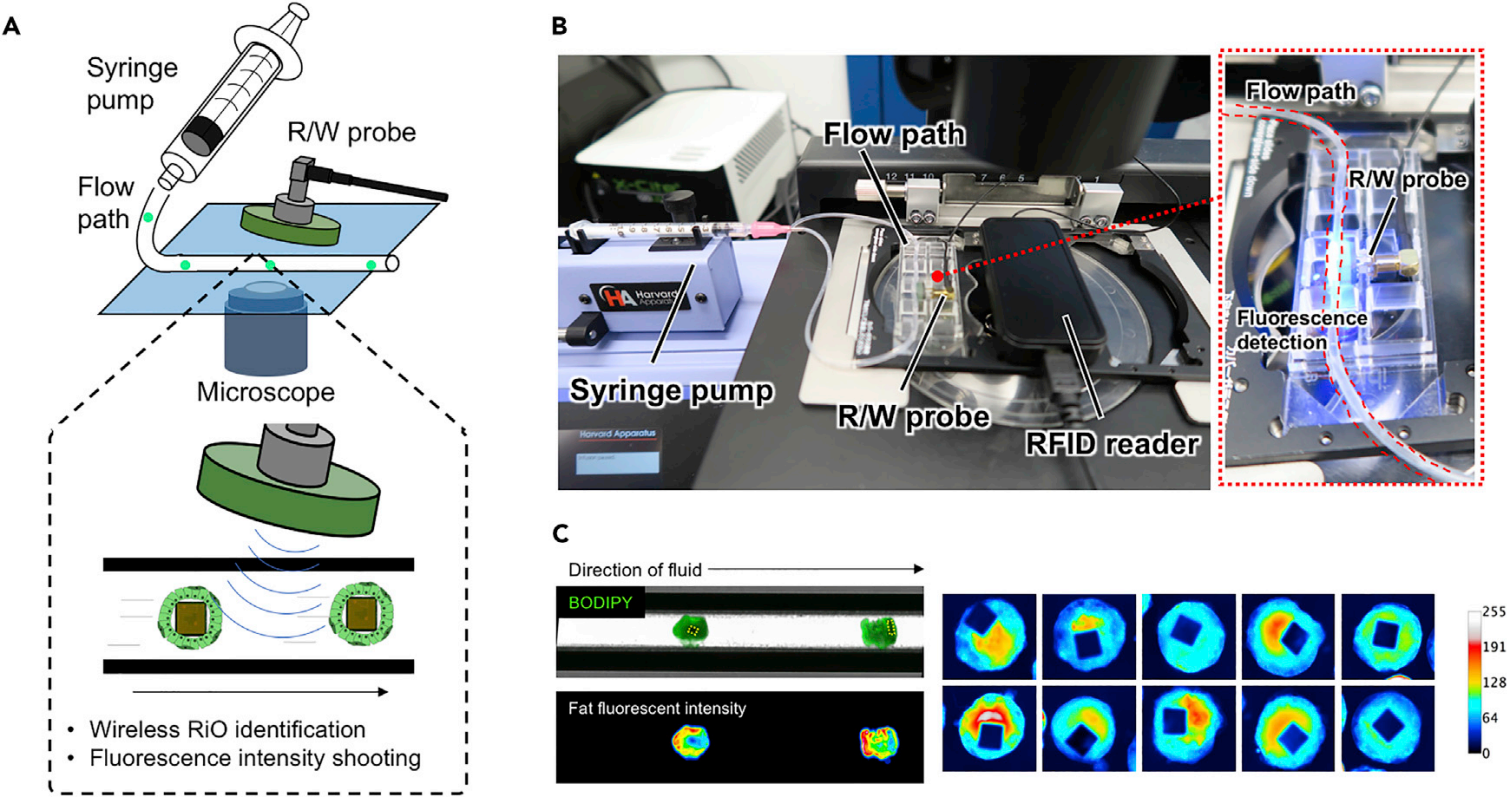
Simultaneous Detection System for Fluorescence Intensity and RFID in RiO (A and B) Measurement workflow and device details for RiO phenotyping. (C) Fluorescent intensity quantification of RiO in the flow path. RiOs were treated with fatty acids, and the amount of lipid accumulation was visualized using a lipid-specific fluorescent dye, BODIPY.

To further identify each RiO, we developed a device to simultaneously measure the fluorescence and detect the RFID chip in one step. The measurement workflow and device details for RiO phenotyping are shown in Figure 3A. Using this detection system, simultaneous Electronic Product Code (EPC) recording and fluorescence imaging of passing RiO are possible through the flow (Figure 3B). This device was composed of a microliter-scale syringe pump, a flow path through which the organoid passes, a detection probe that recognizes RFID, and a fluorescence microscope. To validate the entire system with RIO, we treated the organoid with fatty acids and then visualized the lipid accumulation in the RiO using a lipid-specific fluorescent dye called BODIPY. Then, RiOs were passed through the flow path using a syringe pump that can adjust the flow rate on a microscale. Subsequently, the RFID signal was detected together with a snapshot of fluorescence intensity (Figure 3C). Thus, we successfully developed a higher-throughput detection device for fluorescence imaging based on phenotyping assay coupled with the RFID-integrated organoids.

Understanding the pathological variations in human diseases with human stem cell models is vital to promote precision medicine and drug screening applications. Community efforts to derive a population human induced pluripotent stem cell (iPSC) library from healthy and diseased donors provide an accessible platform to study human gene expressional variation, such as gene expression quantitative trait loci (eQTL) (Kilpinen et al., 2017). However, head-to-head manual comparison among larger numbers of different lines is completely inefficient and unrealistic, and, more importantly, the organoid-level phenotyping method remains to be developed. To circumvent this challenge, we used the RiO-based approach so as to detect the specific donor from pools after identifying a notable phenotype through an initial screen.

**Figure 4:**
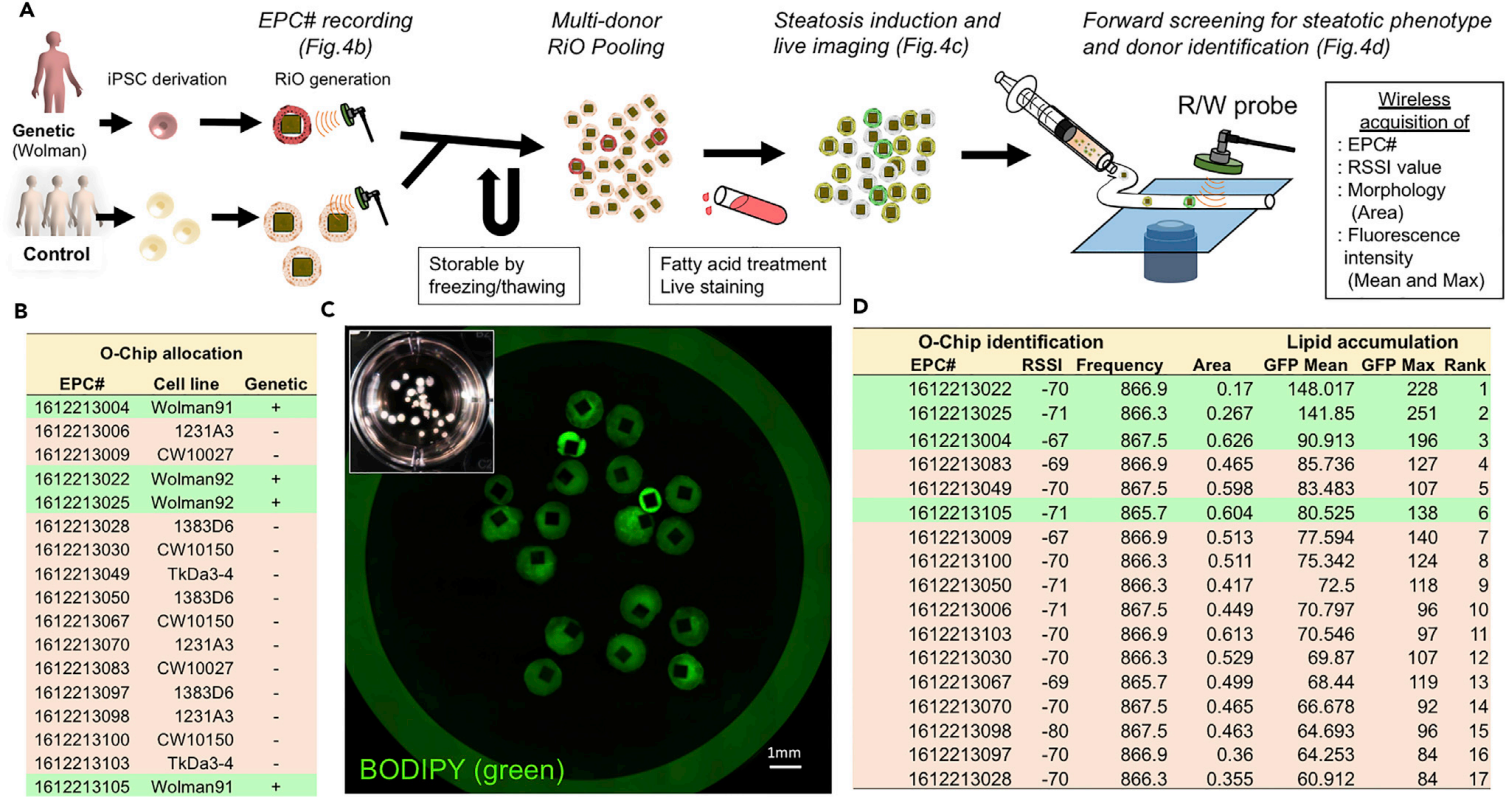
Forward Cellomic Screen of Multi-Donor-Derived RiO Pool (A) The RiO generated from seven different iPSC lines, including two from patients with Wolman disease. The RiOs were then pooled together in one culture and treated with fatty acid. The amount of accumulated lipids was quantified by fluorescent imaging as a method to screen for steatosis. (B) EPC number allocation per specific donor before freezing. Green highlighted cell lines were two Wolman disease iPSCs, and the other pink ones were five control iPSCs. (C) A fluorescent image of pooled RiO after steatosis induction demonstrating lipid accumulation using BODIPY. RiOs derived from different donors have varying lipid accumulation. Identification of each individual RiO from different iPSC lines is not possible by visual inspection. Scale bars for RiO, 1 mm. (D) Quantification of individual RiO fluorescence intensity, followed by wireless detection of EPC# in RiO by RFID reader. RiOs derived from a patient with Wolman disease accumulate the most lipids, relative to five other control iPSC lines.

As a proof-of-principle phenotypic screen, we specifically generated a small pool of frozen RiOs (Figures 4A, 4B, and S7). We selected seven different donor-derived iPSCs that included two patients with a monogenic form of steatohepatitis, called Wolman disease. Wolman disease is caused by the absence of lysosomal acid lipase (LAL), and hepatocytes from patients with Wolman disease have heavy lipid accumulation, so measuring lipid accumulation of hepatocytes can provide an effective screen for steatosis. We confirmed prominent steatosis progression in Wolman-derived LO after exposure to free fatty acids (Figure S8). Similarly, BODIPY live imaging was conducted against the pooled RiO (Figure 4C). Fluorescent microscopy imaging confirmed significantly higher intensity in specific RiO versus others (Figure 4D). We identified each RiO donor by its RFID chip and discovered that these higher-intensity RiO (indicative of increased steatosis) corresponded to Wolman disease-derived iPSC organoids (Figure 4D). Thus, these RiOs have successfully recapitulated human, genetic-based, steatohepatitis pathology by reflecting human genetic disease. More importantly, a RiO-based pooling approach will be an efficient way to determine individualized phenotypes in a high-throughput setting.

## Discussion

We herein present a strategy to track a spectrum of organoid phenotypes by integrating a digital miniaturized RFID into biological tissues. The key to success is to ensure that the organoids maintain their native function. As the collective cells experience aggregation via a self-assembling cavitation process, the micro-chips seamlessly locate into the intra-lumen of organoids without compromising native structures, preventing any tissue damage or destruction. Strikingly, the chip-integrated organoids underwent freezing and thawing cycles with no obvious loss of viability and would, therefore, greatly facilitate the banking efforts of multiple donor-derived organoids. Given that the miniaturization of digital devices, including biosensors (Zhong et al., 2016), robotics (Nelson et al., 2010), and cameras (Liu et al., 2015), is rapidly evolving (Bergeles and Yang, 2014, Chai et al., 2016, Sitti et al., 2015), the overall strategy exemplified with RFID will enable the development of new integral approaches for the sensing, management, and intervention of biological tissues.

About 5,000+ pluripotent stem cell lines have been established and deposited into emerging biobanks, such as HipSci, EBiSC, WiCell, Coriell, RIKEN, and NYSCF. A recently proposed “cellomics” approach, in which population stem cell array is coupled to the assessment of the phenotypic variabilities of each line, is a highly attractive approach to screen and define the cellular and molecular basis for human variations, such as expression (eQTL)-based association studies (Kilpinen et al., 2017). However, conventional laboratory-scale protocols are essentially inefficient and not equipped to perform large comparative analyses, as culturing each cell line separately is costly and labor intensive. In contrast, the current RFID market price is around US$0.1–0.2. Even when considering that large numbers of stem cell or primary cell lines are used, the overall cost for analysis is by far inexpensive than available cutting-edge single-cell genomics approaches for identifying individual in a pooled condition. The RFID-based strategy is a cost-effective and, therefore, scalable approach to enable forward donor identification.

More importantly, organoid-level phenotyping, and not just transcriptomic analysis, is particularly demanding for drug screening or precision medicine applications. RiO-based phenotyping at the organoid level, we believe, is a different class of approach to determine larger scale phenotype, not just a transcriptomic characterization. By taking advantage of donor identifiable organoids and expanding on existing concepts, we propose a “forward cellomics” approach as a potential strategy to determine the personalized phenotypes in a pooled condition. Forward genetics is a known scalable approach of studying genetic impacts on phenotype as a counter strategy to reverse genetics, which determines the function of a gene by analyzing the phenotypic effects of altered DNA sequences. Likewise, a forward cellomics approach would first determine phenotypic variations by existing screen methods and then study the causal genetic and cellular bases for pronounced differences. Our data suggested the possibility of this “forward” phenotyping methods, at least for determining the monogenic basis for pathological phenotype of steatohepatitis. By pooling the organoids in the same well, significant cost reduction will be expected besides enhanced reproducibility of the experiments. Further efforts are needed to maximize forward cellomics potential, coupled to developing high-throughput organoid phenotyping assays (Dekkers et al., 2013), all of which could be extended to other organoid systems or diseases. Nevertheless, the presented methodology is scalable and time- and cost-efficient. Digitalized organoids will potentially facilitate the *in vitro* study of an extensive population cohort by examining variable disease phenotypes, drug safety, and efficacy.

## Methods

All methods can be found in the accompanying Transparent Methods supplemental file.

## Supplemental Information

Document S1. Transparent Methods, Figures S1–S8, and Table S1
