# Supplementary material for: Digitalized Human Organoid for Wireless Phenotyping
Source: iScience. 2018 May 31;4:294–301. doi: 10.1016/j.isci.2018.05.007 (PMC6147234; doi:10.1016/j.isci.2018.05.007)
Supplement: Document S1. Transparent Methods, Figures S1–S8, and Table S1 [file mmc1.pdf]

ISCI, Volume *4*

Supplemental Information

Digitalized Human Organoid for Wireless Phenotyping

Masaki Kimura, Momoko Azuma, Ran-Ran Zhang, Wendy Thompson, Christopher N. Mayhew, and Takanori Takebe

TRANSPARENT METHODS

Maintenance of PSCs Two human iPSC lines, 1231A3 and 1383D6 were obtained from Kyoto University. The TkDA3-4 line was kindly provided by K. Eto and H. Nakauchi. The CW10027 and CW10150 lines were obtained from the NINDS iPSC Repository at Coriell Institute. The Wolman disease Wolman91 and Wolman92 iPSC lines were reprogrammed at Cincinnati Children's Hospital Medical Center (CCHMC) Pluripotent Stem Cell Facility. Human iPSC lines were maintained as described previously (Takebe et al., 2015; Takebe et al., 2014). Undifferentiated human iPSCs were maintained on feeder-free conditions in mTeSR1 medium (StemCell technologies, Vancouver, Canada) on plates coated with Matrigel (Corning Inc., NY, USA) at 1/30 dilution at 37°C in 5% CO2 with 95% air.

Definitive endoderm induction. Human iPSCs differentiation into definitive endoderm was done using previously described methods with slight modifications [26]. In brief, colonies of human iPSCs were isolated in Accutase (Thermo Fisher Scientific Inc., MA, USA) and 150,000 cells/mL were plated on Matrigel coated tissue culture plates (VWR Scientific Products, West Chester, PA). Medium was changed to RPMI 1640 medium (Life Technologies) containing 100 ng/mL Activin A (R&D Systems, MN, USA) and 50 ng/mL bone morphogenetic protein 4 (BMP4; R&D Systems) on day 1, 100 ng/mL Activin A and 0.2 % fetal calf serum (FCS; Thermo Fisher Scientific Inc.) on day 2 and 100 ng/mL Activin A and 2% FCS on day 3. Day 4-6 cells were cultured in Advanced DMEM/F12 (Thermo Fisher Scientific Inc.) with B27 (Life Technologies) and N2 (Gibco, CA, USA) containing 500 ng/ml fibroblast growth factor 4 (FGF4; R&D Systems) and 3 uM CHIR99021 (Stemgent, MA, USA).

Cells were maintained at 37°C in 5% CO2 with 95% air and the medium was replaced every day. Spheroids appeared on the plate at day 7 of differentiation.

RFID chip incorporated liver organoid (RiO) generation. At day 7, definitive endoderm cells were dissociated to single cells in Accutase (Thermo Fisher Scientific Inc., Waltham, MA, USA), spun down and quickly resuspended at the desired concentration in differentiation medium ($5.0X10^5$ cells per 100 μL). Differentiation medium was Advanced DMEM/F12 with B27, N2, 2 μM retinoic acid (RA; Sigma, MO, USA) and 10μM ROCK inhibitor Y-27632(R&D Systems, Minneapolis, MN). Cells were loaded into 96-well ultra-low attachment (ULA) U bottom plates (Corning, Acton, MA, USA) in 200μL volume respectively. After seeding the cells, the ultra-compact RFID chip (SK-Electronics Co., Ltd., Japan) was placed in each well and centrifuged for 1 minute at 130g. Ultra-compact RFID chips are able to purchase from SK-Electronics website(http://www.sk-el.co.jp/sales/rfid/en/index.html). Plates were then incubated overnight at 37°C, 5% CO2. The following day, the medium was replaced with freshly prepared differentiation medium without ROCK inhibitor. The medium was changed every day. After 3 days of culture, the medium was replaced with Hepatocyte Culture Medium (HCM; Lonza, MD, USA) with 10 ng/mL hepatocyte growth factor (HGF; PeproTech, NJ, USA), 0.1 uM Dexamethasone (Dex; Sigma) and 20 ng/mL Oncostatin M (OSM; R&D Systems). After 10 -15 days of culture, human iPSC derived RiOs were detached, collected and analyzed.

Albumin ELISA. To measure the albumin secretion level of RiO, RiO were seeded and cultured on 24-well ULA plates (Corning). To define the exact number of RiO in each well, the RiOs were captured on the BZ-X710 Fluorescence Microscope

(Keyence, Osaka, Japan). The culture supernatants were collected at a 24hr time point after the initial culture and stored at - 80°C until use. The supernatant was centrifuged at 1,500 rpm for 3 min to pellet debris, and the resulting supernatant was assayed with the Human Albumin ELISA Quantitation Set (Bethyl Laboratories, Inc., TX, USA) according to the manufacturer's instructions. Significance testing was conducted by Student's *t*-test.

Whole mount immunofluorescence. RiO were fixed for 30 min in 4 % paraformaldehyde and permeabilized for 15 min with 0.5% Nonidet P-40. RiO were washed by 1x PBS three times and incubated with blocking buffer for 1 h at room temperature. RiO were then incubated with primary antibody; anti-albumin antibody (Abcam) and anti- hepatocyte nuclear factor 4 alpha antibody (Santa Cruz) overnight at 4 °C. RiO were washed by 1 x PBS and incubated in secondary antibody in blocking buffer for 30 min at room temperature. RiO were washed and mounted using Fluoroshield mounting medium with DAPI (Abcam). The stained RiO were visualized and scanned on a Nikon A1 Inverted Confocal Microscope (Japan) using 60× water immersion objectives.

RNA isolation, RT–qPCR. RNA was isolated using the RNeasy mini kit (Qiagen, Hilden, Germany). Reverse transcription was carried out using the High-Capacity cDNA Reverse Transcription Kit (Thermo Fisher Scientific Inc.) according to manufacturer's protocol. qPCR was carried out using TaqMan gene expression master mix (Applied Biosystems) on a QuantStudio 3 *Real-Time PCR* System (Thermo Fisher Scientific Inc.). All primers and probe information for each target gene was obtained from the Universal ProbeLibrary Assay Design Center

(https://qpcr.probefinder.com/organism.jsp). Significance testing was conducted by Student's *t*-test.

Rhodamine123 and Cholyl-Lysyl-Fluorescein transport assay. RiO were incubated with 100μM of Rhodamine 123(Sigma) and 5uM of Cholyl-Lysyl-Fluorescein (CLF, Corning Incorporated) for 10 minutes at 37°C. Next, RiO were washed three times with PBS. Images were captured on the KEYENCE BZ-X710 Fluorescence Microscope (Keyence).

Live-cell imaging of lipid accumulation. RiO from 6 donors were pooled in ULA 24-well plates (Corning) and subjected to live-cell staining. For lipid accumulation, RiOs were treated with 100μM of oleic acid (Sigma) for 24hr at 37°C. Quantitative estimation of lipid accumulation was performed by BODIPY® 493/503 (Thermo Fisher Scientific Inc.). Images were captured on the KEYENCE BZ-X710 Fluorescence Microscope (Keyence) and fluorescence intensity was measured by ImageJ 1.48k software (Wayne Rasband, NIHR, USA, http://imagej.nih.gov/ij).

Cryopreservation and thawing of RiO. For RiO cryopreservation, RiO were washed three times with PBS and resuspended in freezing medium. The following freezing mediums were used: CELLBANKER® 1(AMS Biotechnology Limited, UK), StemCell Keep™(Abnova), PBS/20%FCS/ ethylene glycol1.8M(EG; Sigma) and DMEM/F12(Gibco)/20%FCS/10% dimethyl sulfoxide (DMSO; Sigma). One to five RiOs were suspended in 400 μL of freezing medium and pipetted into a cryovial. CELLBANKER® 1 and StemCell Keep were used according to the manufacturer's protocol. PBS/FCS/ EG and DMEM/F12/FCS/DMSO conditions were directly frozen

at -80 °C. For thawing, frozen RiOs were quickly thawed and washed with culture medium. Thawed RiOs were cultured and analyzed.

SUPPLEMENTAL FIGURES AND LEGENDS

						After processing		
		#	EPC# assignment		EPC#	RSSI	Frequency	Antenna
Freezing	RT	1	1612213035	→	1612213035	-70	866.9	0
		2	1612213004	→	1612213004	-70	866.9	0
		3	1612213005	→	1612213005	-66	866.9	0
	4°C	1	1612213081	→	1612213081	-69	866.3	0
		2	1612213047	→	1612213047	-70	865.7	0
		3	1612213087	→	1612213087	-69	866.9	0
	-20°C	1	1612213045	→	1612213045	-68	867.5	0
		2	1612213080	→	1612213080	-71	866.9	0
		3	1612213095	→	1612213095	-69	865.7	0
	-80°C	1	1612213102	→	1612213102	-71	866.3	0
		2	1612213086	→	1612213086	-70	865.7	0
		3	1612213097	→	1612213097	-70	866.3	0
	-196°C	1	1612213022	→	1612213022	-69	865.7	0
Autoclaving	121°C	1	1612213026	→	1612213026	-67	865.7	0
		2	1612213037	→	1612213037	-69	866.9	0
		3	1612213057	→	1612213057	-69	865.7	0
Acidic environment	pH1.0	1	1612213026	→	1612213026	-67	865.7	0
		2	1612213037	→	1612213037	-69	866.9	0
		3	1612213057	→	1612213057	-69	865.7	0
	pH2.0	1	1612213026	→	1612213026	-67	865.7	0
		2	1612213037	→	1612213037	-69	866.9	0
		3	1612213057	→	1612213057	-69	865.7	0
	pH3.0	1	1612213026	→	1612213026	-67	865.7	0
		2	1612213037	→	1612213037	-69	866.9	0
		3	1612213057	→	1612213057	-69	865.7	0
	pH4.0	1	1612213026	→	1612213026	-67	865.7	0
		2	1612213037	→	1612213037	-69	866.9	0
		3	1612213057	→	1612213057	-69	865.7	0
	pH5.0	1	1612213026	→	1612213026	-67	865.7	0
		2	1612213037	→	1612213037	-69	866.9	0
		3	1612213057	→	1612213057	-69	865.7	0
	PH6.0	1	1612213026	→	1612213026	-67	865.7	0
		2	1612213037	→	1612213037	-69	866.9	0
		3	1612213057	→	1612213057	-69	865.7	0

Supplemental Table. Tolerance of RFID (Related to Figure2)

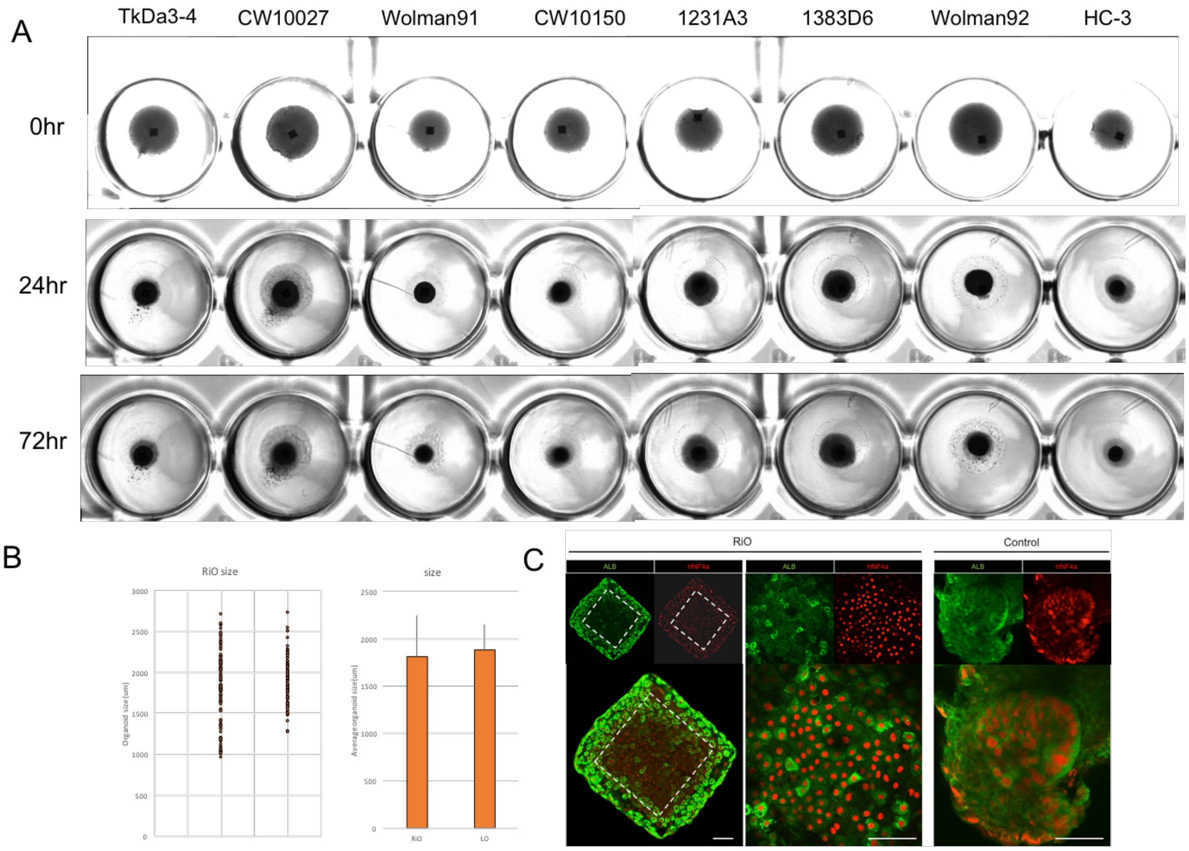

Supplemental Fig.1. Reproducible RiO generation from multiple donor-derived iPSC liver organoids. (Related to Figure2) **A,** RiO shows similar morphological and immunological profiles to HLO. **B**, morphology of RiO and control HLO. **C**, immunostaining of RiO and control HLO. Middle panel shows higher magnification of central part of RiO shows ALB and HNF4A expression, although thickness of the tissues weaken the fluorescence intensity.

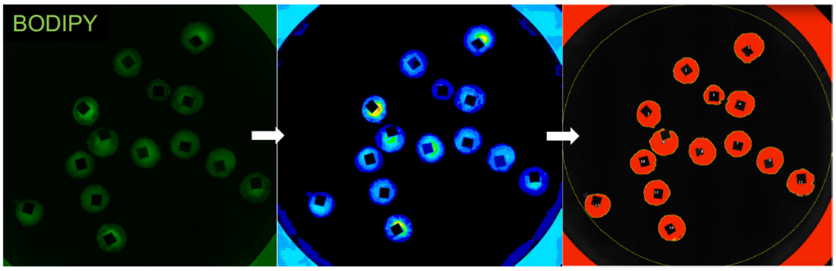

RiO pool with BODIPY staining

BODIPY intensity converted brightness and darkness in an image into numbers by ImageJ LUT.

The fluorescence intensity of each organoid was measured using imageJ.

	GFP intensity			
#	Mean	Median	Max	Area(px)
1	60.299	53	145	25400
2	48.219	47	78	19301
3	35.571	35	207	9217
4	49.282	48	114	22532
5	76.475	66	178	18724
6	59.615	55	118	21846
7	53.493	54	84	20378
8	62.933	60	115	23166
9	54.792	53	84	21817
10	52.604	51	89	18030
11	46.236	42	104	20957
12	52.361	54	76	19024
13	56.22	54	96	16920
14	59.999	52	151	21042

Supplemental Fig.2. Measurement workflow of fat fluorescent intensity in RiO.

(Related to Figure2)

The fat accumulation capacity of RiO was studied using the fatty acid treatment and lipid dye BODIPY® 493/503 for lipids.

FAS 100μM treatment 37˚C 30min
Fe (II) detection(FeRhoNox-1 5μM, 1hr)

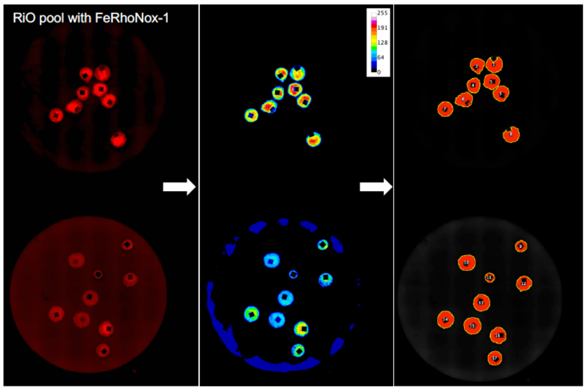

FeRhoNox-1 intensity converted brightness and darkness in an image into numbers by ImageJ LUT.

The fluorescence intensity of each organoid was measured using imageJ.

	RFP intensity			
RiO#	Mean	Median	Max	Area(px)
1	115.818	117	185	2.831
2	123.132	123	221	2.074
3	141.833	147	254	2.539
4	112.79	111	210	1.947
5	133.389	139	224	2.443
6	128.555	126	222	2.534
7	120.284	123	192	2.348
8	132.669	139	216	2.742
9	90.066	90	255	1.097
10	72.519	72	104	2.908
11	60.58	60	86	0.461
12	77.431	76	225	1.937
13	73.376	73	113	2.855
14	93.705	96	211	2.907
15	71.634	72	99	3.025
16	109.561	110	186	2.631
17	86.484	87	150	1.921

Supplemental Fig.3. Detection of iron accumulation in RiO. (Related to Figure2)

Iron accumulation capacity was studied using the ammonium iron sulfate (FAS) treatment and Fe dye FeRhoNox® 540/575.

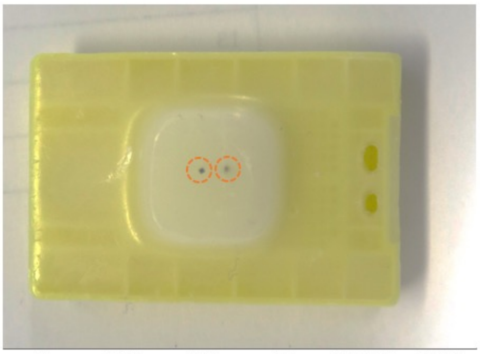

#	Count	EPC	RSSI	Frequency	Antenna
1	37	1612213096	-69	866.3	0
2	18	1612213013	-55	866.9	0

Supplemental Fig.4. Wireless detection of paraffin embedded RiO. (Related to Figure2)

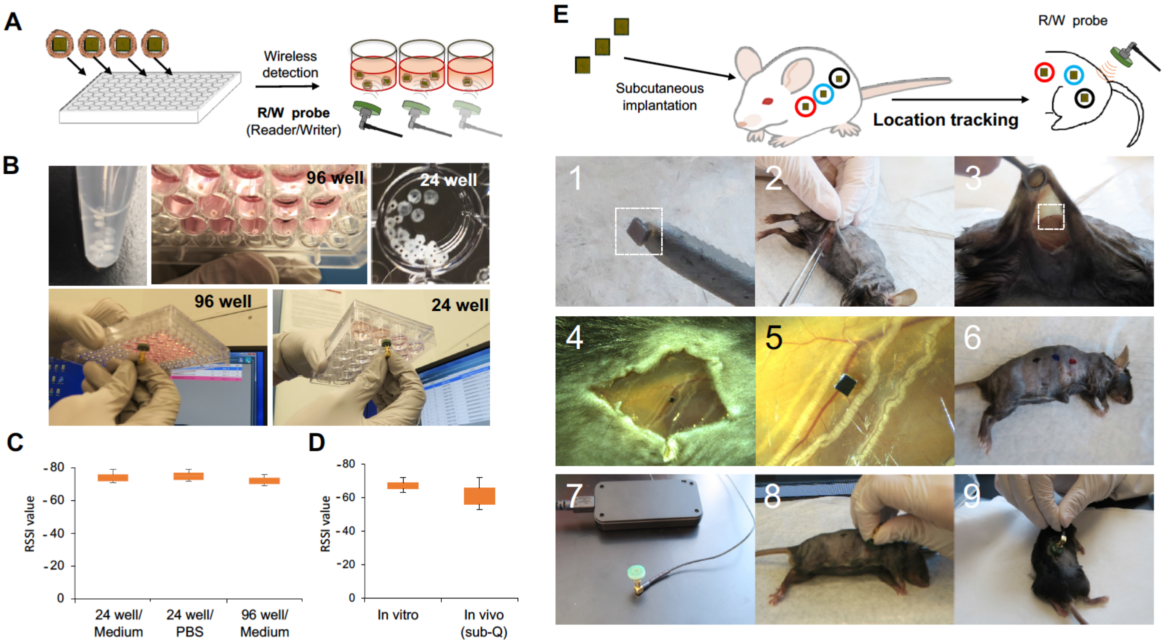

Supplementary Fig.5 Wireless identification of RiO in vitro and in vivo and in vivo. (Related to Figure2) (**A, B**) Scheme of RFID identification in RiO. Generated RiO were placed into 96 and 24 well plastic plates. The RFID signal was detected by the detection probe from the bottom of the plate. (**C**) Measurement of RSSI value under various media conditions. (**D)** Measurement of RSSI value in vitro and in vivo. (**E**) Tracking of O-Chip location after implantation. After embedding the O-Chip under mouse skin, RFID signal was measured with a reader directly over the mouse's skin. All animal experiments were performed with the approval of the Institutional Animal Care and Use Committee of CCHMC (protocols 2015-0085).

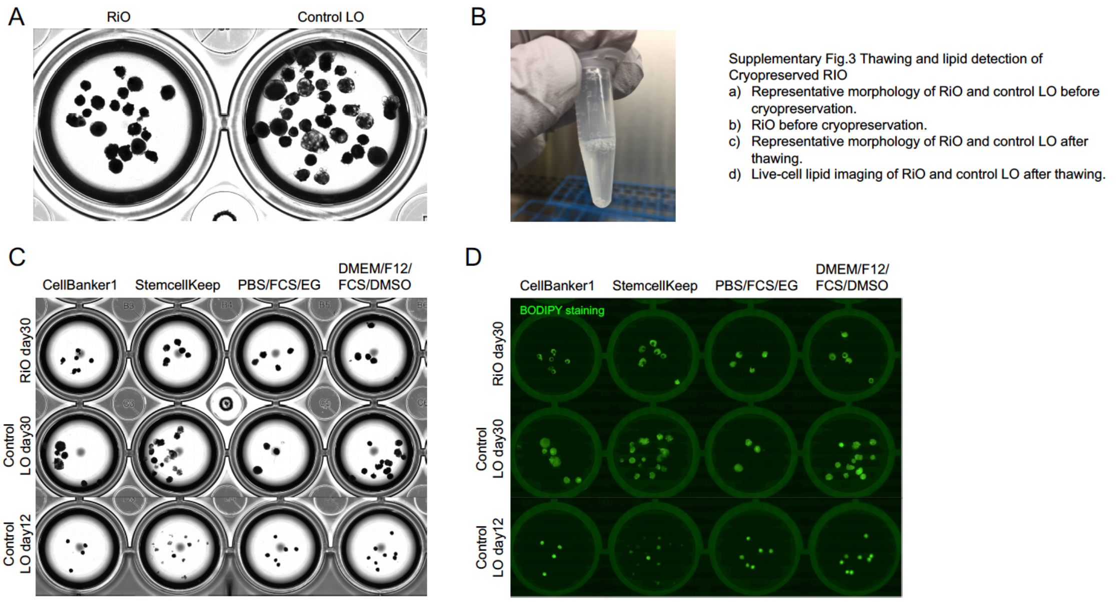

Supplementary Fig.6 Thawing and lipid detection of cryopreserved RiO.

(Related to Figure2) **(A)** Representative morphology of RiO and control LO before cryopreservation. (**B**) RiO before cryopreservation. (**C**) Representative morphology of RiO and control LO after thawing. (**D**) Live-cell lipid imaging of RiO and control LO after thawing.

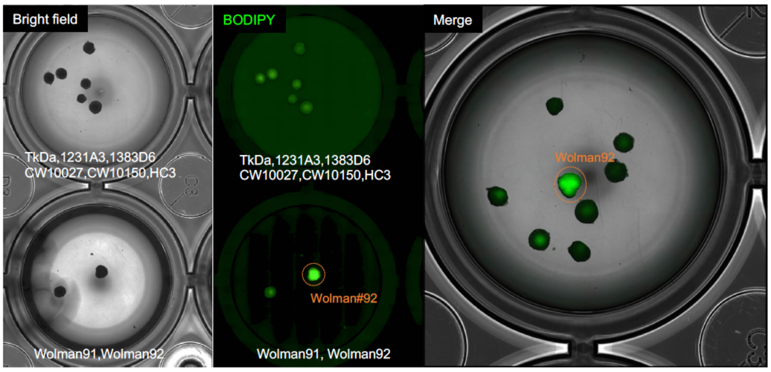

Supplemental Fig.7. RiO phenotype is similar to normal iPSC LO. (Related to Figure4)

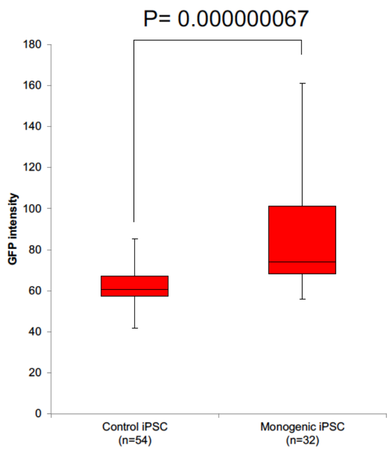

Monogenic disease iPSC line	
WD90(n=16)	LAL deficiency
WD91(n=7)	LAL deficiency
WD92(n=9)	LAL deficiency

Healthy/polygenic disease iPSC line	
1231A3(n=6)	Healthy
1383D6(n=5)	Healthy
CW10027(n=9)	Healthy
VW10077(n=7)	NASH
CW10150(n=7)	NASH
CNS-1(n=7)	Crigler–Najjar syndrome
HC-3lc2(n=13)	Hemochromatosis

Supplemental Fig.8. Difference in lipid accumulation between healthy iPSCs and monogenic iPSC line. (Related to Figure4)
